# Cutaneous Events After Recombinant Zoster Vaccination and Shoulder Injury Related to Zoster Vaccine Administration: A Narrative Review

**DOI:** 10.3390/jcm15207962

**Published:** 2026-10-15

**Authors:** Carlo Ciccullo, Arturo Ciccullo, Sonia Farah, Claudia Bevilacqua, Marco Grassi, Luca Farinelli, Edoardo De Simoni, Elisa Molinelli, Oriana Simonetti, Simone Domenico Aspriello, Antonio P. Gigante

**Affiliations:** 1 Clinical Orthopaedics, Department of Clinical and Molecular Sciences, Politecnica University of Marche, 60126 Ancona, Italy; carlo.ciccullo19@gmail.com (C.C.); claudia.bevilacqua@ospedaliriuniti.marche.it (C.B.); marco.grassi7@gmail.com (M.G.); farinelli.luca92@gmail.com (L.F.); a.p.gigante@staff.univpm.it (A.P.G.); 2 Department of Medicine, Infectious Diseases Unit, San Salvatore Hospital, 67100 L’Aquila, Italy; arturo.ciccullo@gmail.com; 3 Rheumatology Unit, Università Politecnica delle Marche, Ospedale “Carlo Urbani”, 60035 Jesi, Italy; sonia.fara91@gmail.com; 4 IRCCS INRCA, 60126 Ancona, Italy; 5 Clinic of Dermatology, Department of Clinical and Molecular Sciences, Politecnica University of Marche, 60126 Ancona, Italy; edodesimoni@hotmail.it (E.D.S.); molinelli.elisa@gmail.com (E.M.); 6 ASD Dental Clinic, 61121 Pesaro, Italy

**Keywords:** Shingrix, recombinant zoster vaccine, rotator cuff disease, SIRVA, shoulder disease

## Abstract

**Background:** Herpes zoster results from the reactivation of varicella–zoster virus (VZV), with incidence increasing with age and immunosuppression. The recombinant zoster vaccine (RZV, Shingrix^®,^ GlaxoSmithKline Biologicals SA, Rixensart, Belgium) has demonstrated high efficacy in preventing herpes zoster and its complications. **Methods:** We conducted a narrative review of English-language publications addressing cutaneous events, musculoskeletal events, and shoulder injury related to vaccine administration following recombinant zoster vaccination. Original studies, pharmacovigilance analyses, case reports, case series, and relevant reviews published up to 31 January 2026, were considered. **Results**: Available evidence on cutaneous and musculoskeletal events following recombinant zoster vaccination was limited and heterogeneous. Reported cutaneous events included inflammatory and hypersensitivity-type reactions, vesicular or bullous eruptions, and episodes of VZV reactivation occurring in temporal proximity to vaccination. In virologically confirmed cases, the detected VZV strains were wild-type, consistent with reactivation of latent wild-type virus. Musculoskeletal symptoms, including arthralgia and myalgia, were more commonly reported but were generally mild and self-limiting. Evidence concerning SIRVA was limited and restricted to a single case report following intramuscular zoster vaccine administration. **Conclusions**: Current evidence supports a favorable safety profile for Shingrix^®^. Cutaneous events are uncommon and heterogeneous, while evidence concerning SIRVA is limited to a single case report following zoster vaccination, precluding estimates of incidence or a product-specific association. Proper injection technique and continued pharmacovigilance remain essential.

## 1. Introduction

Varicella–zoster virus (VZV) causes varicella during primary infection and subsequently remains latent in sensory ganglia. Reactivation of latent VZV results in herpes zoster (HZ), which typically presents as a painful vesicular eruption with a dermatomal distribution [1,2,3]. The incidence of HZ increases with age and immunosuppression because of declining VZV-specific cell-mediated immunity. Postherpetic neuralgia is the most common complication of HZ and may substantially impair quality of life, particularly in older adults [2,4]. Other complications include ophthalmic, neurological, cutaneous, and visceral involvement. HZ may also recur, especially in immunocompromised individuals, although recurrence may also occur in immunocompetent persons [5,6,7]. Given the clinical burden of HZ and its complications, vaccination represents the principal preventive strategy.

Zoster vaccine live (ZVL, Zostavax^®^, Merck Sharp & Dohme (MSD), Rahway, NJ, USA) was approved in 2006, but its efficacy decreases with increasing age and wanes over time [8]. The principal differences between ZVL and the recombinant zoster vaccine (RZV, Shingrix^®^) are summarized in Table 1.

The recombinant zoster vaccine (RZV; Shingrix^®^, GSK) was initially approved by the US Food and Drug Administration in 2017 for the prevention of HZ in adults aged 50 years or older. Its regulatory indication was subsequently expanded to adults aged 18 years or older who are or will be at increased risk of HZ because of immunodeficiency or immunosuppression resulting from disease or therapy. In the United States, the Advisory Committee on Immunization Practices recommends two doses of RZV for immunocompetent adults aged 50 years or older and for adults aged 19 years or older who are or will be immunodeficient or immunosuppressed [9,10]. Shingrix^®^ is a non-live vaccine containing 50 µg of VZV glycoprotein E (gE) and the AS01B adjuvant system, which comprises 50 µg of 3-O-desacyl-4′-monophosphoryl lipid A and 50 µg of QS-21. Glycoprotein E is abundantly expressed in VZV-infected cells and represents a major target of humoral and cell-mediated immune responses [11,12]. The components of AS01B act synergistically to enhance innate and adaptive immune responses and promote interferon-γ production [13,14,15].

Shingrix^®^ is administered intramuscularly into the deltoid muscle as a two-dose series, with the second dose generally administered 2–6 months after the first. The complete series induces robust humoral and cell-mediated immune responses across the age groups studied, with sustained immune responses also demonstrated in older adults [16,17]. Shingrix^®^ commonly causes transient local reactogenicity, including pain, erythema, and swelling at the injection site. SIRVA is a distinct clinical condition characterized by persistent shoulder pain and restricted range of motion, typically beginning within 48 h after vaccination in individuals without pre-existing shoulder symptoms [18,19]. It is primarily related to improper injection technique and inadvertent deposition of vaccine material into structures adjacent to the deltoid muscle rather than to the biological properties of a specific vaccine. VZV reactivation has also been reported in temporal association with other vaccines, particularly during the COVID-19 vaccination campaign. Case reports and observational data have suggested a possible short-term association, although temporal proximity alone does not establish causality, and these findings must be interpreted in light of the background incidence of HZ and potential confounding factors [20,21]. An agent-based modelling study also examined whether reduced circulation of wild-type VZV following childhood varicella vaccination could affect HZ epidemiology by limiting exogenous immune boosting. However, this remains a population-level hypothesis and should not be conflated with vaccine-induced viral reactivation or with the safety profile of RZV [22]. This narrative review summarizes the available evidence on cutaneous and musculoskeletal events following Shingrix^®^ vaccination and discusses SIRVA after zoster vaccine administration, with particular attention to expected reactogenicity, pharmacovigilance signals, temporal associations, and limitations of causal inference.

## 2. Materials and Methods

We conducted a narrative review supported by a structured literature search. PubMed/MEDLINE and Google Scholar were searched for English-language publications available through 31 January 2026. Search concepts combined terms related to recombinant zoster vaccination, including “recombinant zoster vaccine”, “RZV”, “Shingrix”, and “zoster vaccination”, with terms related to the outcomes of interest, including “cutaneous reaction”, “skin manifestation”, “vesicular eruption”, “bullous eruption”, “herpes zoster reactivation”, “arthralgia”, “arthritis”, “musculoskeletal adverse event”, “shoulder injury related to vaccine administration”, “SIRVA”, “vaccine safety”, and “post-marketing surveillance”.

Original clinical studies, observational studies, pharmacovigilance analyses, case series, case reports, and relevant reviews were considered when they provided clinically relevant information on cutaneous or musculoskeletal manifestations temporally reported after recombinant zoster vaccination or on the clinical interpretation of SIRVA. Reference lists of relevant publications were also examined to identify additional sources. The evidence was organized narratively according to event category and study design, with particular attention to the method of adverse-event ascertainment, laboratory confirmation, time to onset, and limitations of causal inference. No formal risk-of-bias assessment or quantitative synthesis was performed.

## 3. Results

The available literature was heterogeneous and included randomized clinical trials, observational studies, pharmacovigilance analyses, active surveillance studies, and case reports. The evidence was organized into four clinically relevant domains: overall safety and expected reactogenicity, cutaneous events, musculoskeletal events and inflammatory-disease flares, and SIRVA. A selected summary of the principal primary studies is provided in Table 2.

### 3.1. Overall Safety of Recombinant Zoster Vaccination

Available clinical trials and observational studies support a generally favorable safety profile for RZV [23,24,25,26,27,28].

In phase 3 clinical trials involving immunocompromised populations, adverse events were mainly related to the expected reactogenicity of the vaccine, including fever, local injection-site reactions, myalgia, chills, and headache [23,28]. In a trial involving patients with hematologic malignancies, 47% of vaccinated individuals reported adverse events within 30 days after vaccination [23].

Similarly, in renal transplant recipients, adverse events were more frequently reported in the vaccine group than in the placebo group, with injection-site pain being the most common symptom [28].

Post-marketing surveillance studies have provided additional information on the safety profile of Shingrix^®^ (GlaxoSmithKline Biologicals SA, Rixensart, Belgium) [29]. Pharmacovigilance analyses have described clinically relevant reported events and potential safety signals, although these data cannot establish incidence or causality. A Medicare-based study identified a small excess risk of GBS during the 42 days after RZV administration in adults aged ≥65 years [30], whereas subsequent active surveillance did not confirm a statistically significant increase in risk [31]. Most spontaneous reports were non-serious and consistent with the known reactogenicity profile of the vaccine [29,32]. Because spontaneous-reporting systems are subject to underreporting, reporting bias, and incomplete clinical information, their findings should be interpreted as reporting patterns or potential safety signals rather than as incidence estimates or evidence of causality.

### 3.2. Cutaneous Events After Recombinant Zoster Vaccination

Among the studies identified, the most detailed data on cutaneous events were reported in an observational study conducted by Orru et al., which analyzed patients presenting with cutaneous events after recombinant zoster vaccination [33].

In this study, 72 patients developed cutaneous events temporally associated with vaccination, most frequently involving thoracic and cervical dermatomes. Cutaneous events occurred after the first dose in 50 cases (69.4%) and after the second dose in 22 cases (30.6%) [33]. The clinical presentation included erythema, macules, papules, vesicles, pustules, hemorrhagic vesicles, and crusts. Vesicular lesions were more commonly observed in patients with positive VZV PCR results, whereas macular lesions were reported mainly in VZV-negative cases [33]. This finding suggests that PCR-negative macular eruptions may represent non-viral inflammatory or delayed hypersensitivity reactions. However, a specific diagnosis of localized toxicoderma cannot be established without supportive clinical or histopathological findings. In most PCR-positive cases, the dermatomal eruptions were ultimately attributed to wild-type VZV rather than vaccine-related infection, suggesting that herpes zoster may occur after vaccination but is not necessarily caused by the vaccine itself [33].

Several biological mechanisms have been proposed to explain VZV reactivation temporally associated with vaccination. These include transient immune modulation following strong adjuvant stimulation, cytokine-mediated inflammatory responses, and temporary alterations in T-cell–mediated viral control. Additional evidence was provided by pharmacovigilance data analyzed by Pirrotta et al., who evaluated spontaneous reports of vesicular and bullous cutaneous eruptions following recombinant zoster vaccination [34]. Among 2423 reports of HZ or related complications, 645 were classified as possible vaccination failures, although only two were laboratory confirmed. Furthermore, 810 reports described non-HZ vesicular or bullous eruptions, most of which were interpreted as delayed hypersensitivity reactions [34]. These spontaneous-reporting data do not provide incidence estimates or establish a causal association with vaccination.

Delayed-type hypersensitivity reactions following vaccination have been previously described and may range from persistent localized pruritic nodules to generalized cutaneous eruptions. The onset of these reactions typically occurs within a few days after vaccination, although delayed presentations several weeks later have also been reported [34,35].

A self-controlled case-series study reported a transient increase in HZ diagnoses shortly after the first dose of RZV in older adults, although the protective effect of vaccination became evident after completion of the two-dose schedule [36]. This finding suggests that early post-vaccination cases may reflect either coincidental temporal association or incomplete protection before completion of the full vaccination schedule.

In addition to observational studies, several case reports have described unusual cutaneous events after Shingrix^®^ vaccination. These include bullous eruptions interpreted as delayed hypersensitivity reactions and vesicular eruptions associated with confirmed VZV reactivation in immunocompromised patients [37,38,39]. For instance, a bullous fixed drug eruption was reported shortly after Shingrix^®^ administration, with histopathological findings suggesting a delayed T-cell–mediated hypersensitivity reaction [37].

In another case report, a patient with a history of hematopoietic stem cell transplantation developed disseminated pustular lesions confirmed as VZV reactivation by PCR analysis several days after vaccination [38].

More recently, a case of HZ occurring within 72 h after Shingrix^®^ vaccination was described in an immunocompetent adult [39].

### 3.3. Musculoskeletal Events

Musculoskeletal events were more frequently reported than clinically defined. In patients receiving immunosuppressive therapy for rheumatologic diseases, arthritis flares following RZV have also been reported [24,27]. In a cohort study including 403 patients, Stevens et al. reported disease flares in 27 patients (6.7%), with all episodes being mild and self-limiting [24]. Similarly, Källmark et al. reported increased rheumatoid arthritis disease activity in 6.5% of patients receiving Janus kinase inhibitors, while adverse events were predominantly mild or moderate [27].

In a manufacturer-based pharmacovigilance analysis of 15,638 spontaneous reports, arthralgia was recorded in 305 reports (2.0%), corresponding to a reporting rate of 3.3 reports per 100,000 distributed doses. These data do not permit estimation of incidence or assessment of causality [29]. Real-world observational studies also reported joint or muscle pain after recombinant zoster vaccination [25,26]. In an Italian cohort study, approximately 15% of vaccinated individuals reported joint or muscle pain, with a higher frequency among female patients [25]. Similarly, another surveillance study reported arthromyalgia as one of the most frequent systemic reactions following vaccination, although all cases were self-limiting [26].

### 3.4. SIRVA After Zoster Vaccination

Evidence specifically addressing SIRVA after zoster vaccination is extremely limited [40]. SIRVA is a recognized complication of vaccine administration that typically results from improper intramuscular injection technique and may manifest as persistent shoulder pain, reduced range of motion, adhesive capsulitis, or subacromial bursitis [19,41,42]. Although SIRVA has been described after other vaccines, including influenza and COVID-19 vaccines [41,42,43], only one report following zoster vaccine administration was identified [40]. Hirai et al. described shoulder pain and magnetic resonance imaging findings consistent with SIRVA after intramuscular zoster vaccination; however, the vaccine product was not specified. Consequently, this report cannot support a product-specific association with Shingrix^®^ or permit estimation of incidence or specific risk factors [40].

**Table 2:** Selected primary studies addressing cutaneous and musculoskeletal events and SIRVA following zoster vaccination.

Study; Country	Design, Population, and Exposure	Events and Timing	Ascertainment	Main Findings
Vink et al., 2020 [28]; multicountry	Phase III RCT; renal-transplant pts, n = 264: RZV, n = 132; placebo, n = 132; two doses	AEs within 7–30 d	Diaries and investigator assessment	More local reactions with RZV; mostly transient; no safety concerns.
Stevens et al., 2020 [24]; United States	Retrospective study; RA/SRD pts, n = 403; 222 received two doses	Flares and AEs within 12 wk of each dose	Medical records and telephone contacts	Flares, 6.7%; other AEs, 12.7%; all mild.
Tavares-Da-Silva et al., 2020 [29]; worldwide	Pharmacovigilance; 15,638 reports; approximately 9.3 million doses distributed	Reactogenicity, cutaneous events, and arthralgia; generally within a few days	Spontaneous reports, MedDRA, and O/E analyses	Most reports were non-serious; no unexpected reporting pattern.
Pirrotta et al., 2021 [34]; worldwide	Pharmacovigilance; 2423 HZ-related and 810 non-HZ cutaneous reports	HZ and vesicular/bullous eruptions after RZV	Safety database, MedDRA, medical review, and O/E analysis	Possible VZV reactivations were fewer than expected; no new safety signal.
Hirai et al., 2022 [40]; Japan	Case report; one pt; zoster vaccine product unspecified	Shoulder pain from day 1, persisting for 6 wk	Clinical examination and MRI	SIRVA attributed to an injection placed too high.
Orru et al., 2023 [33]; Germany	Multicenter study; 72 pts: 50 after dose 1 and 22 after dose 2	Cutaneous eruptions within 28 d	Clinical assessment, PCR, and VZV genotyping	Twenty-seven wild-type VZV-positive and 45 VZV-negative cases.
Costantino et al., 2024 [25]; Italy	Observational study; 271 frail pts; 254 received two doses	Local and systemic AEFIs after each dose	Questionnaire and telephone follow-up	AEFIs after dose 1, 37%; after dose 2, 41%; mostly mild.
Källmark et al., 2024 [27]; Sweden	Non-randomized study; RA pts receiving JAKi, n = 91; controls, n = 51; two doses	Reactogenicity and RA activity after each dose	Questionnaires, clinical assessment, and medical records	Mostly mild/moderate AEs; increased RA activity by 6.5%.
Stefanizzi et al., 2024 [26]; Italy	Active surveillance; 538 high-risk pts; 1031 doses	AEFIs within 7 d; flares within 3 mo	Patient diaries and follow-up	Most AEFIs began within 24 h; arthromyalgia, 5.72/100 follow-ups.
Shu et al., 2025 [32]; United States	VAERS analysis; 66,849 reports	General, cutaneous, musculoskeletal, and neurological reports; median onset, 1 d	MedDRA and disproportionality analysis	Most reports were non-serious; incidence and causality could not be established.

Abbreviations: AE, adverse event; AEFI, adverse event following immunization; d, days; HZ, herpes zoster; JAKi, Janus kinase inhibitor; MedDRA, Medical Dictionary for Regulatory Activities; mo, months; MRI, magnetic resonance imaging; O/E, observed-to-expected; PCR, polymerase chain reaction; RA, rheumatoid arthritis; RCT, randomized controlled trial; RZV, recombinant zoster vaccine; SIRVA, shoulder injury related to vaccine administration; SRD, systemic rheumatic disease; VAERS, Vaccine Adverse Event Reporting System; VZV, varicella-zoster virus; wk, weeks.

## 4. Discussion

The available evidence comprises clinical trials, observational studies, pharmacovigilance analyses, and case reports, each with distinct strengths and limitations. Clinical trials provide the most reliable assessment of common reactogenicity but are generally not powered to detect very rare events. Observational studies offer greater clinical characterization but remain susceptible to confounding, whereas spontaneous-reporting systems can identify potential safety signals but cannot establish incidence or causality. Case reports may identify unusual clinical presentations but cannot determine product-specific risk. Within these limitations, the available evidence supports a favorable safety profile for Shingrix^®^, and the findings of this review do not justify changes to current vaccination practice [28,29,32].

Cutaneous events represent a heterogeneous group of manifestations that may occur after vaccination [33,34]. Available evidence suggests that these manifestations may include inflammatory or hypersensitivity reactions and episodes of wild-type VZV reactivation occurring in temporal proximity to vaccination [33,34]. In the study by Orru et al., PCR-positive dermatomal eruptions involved wild-type VZV, excluding vaccine-derived infection in the tested cases [33]. The pharmacovigilance findings reported by Pirrotta et al. identified reporting patterns but did not establish incidence or causality [34].

A self-controlled case-series study reported a transient increase in HZ diagnoses during the weeks following the first RZV dose, whereas protection became evident after completion of the two-dose schedule [36]. These findings should be interpreted with caution because temporal association does not necessarily imply causality. Early post-vaccination diagnoses may reflect the background incidence of HZ among populations selected for vaccination because of older age or underlying comorbidities.

For vaccinators, expected injection-site reactogenicity should be distinguished from SIRVA. Transient deltoid pain without persistent functional limitation is consistent with common reactogenicity, whereas SIRVA typically begins within 48 h after vaccination and is characterized by persistent pain and restricted range of motion in a previously asymptomatic shoulder [18,41]. The differential diagnosis includes rotator-cuff disease, subacromial bursitis, adhesive capsulitis, septic arthritis, and cervical radiculopathy [41,42]. Persistent or severe pain, substantial limitation of movement, weakness, fever, swelling, or neurological symptoms should prompt further clinical evaluation and, when appropriate, imaging [41,42]. Correct anatomical landmarking, appropriate needle selection, and injection into the central deltoid region remain the principal preventive measures [19,42].

Evidence specifically addressing SIRVA after zoster vaccination remains extremely limited. The only identified case report described SIRVA after intramuscular zoster vaccine administration, but the vaccine product was not specified; therefore, it cannot support a product-specific association with Shingrix^®^ [40]. The apparent scarcity of cases may reflect true rarity, but under-recognition and underreporting are also plausible. Post-vaccination shoulder pain may be coded nonspecifically as injection-site pain, arthralgia, bursitis, or shoulder pain without assessment of the diagnostic criteria for SIRVA [41,42].

Pharmacovigilance reports should document the side of vaccination, time to onset, symptom duration, range-of-motion findings, previous shoulder disease, injection site and technique, imaging findings, and final diagnosis. Future studies should apply standardized case definitions and use denominator-based surveillance to distinguish expected reactogenicity from true SIRVA and permit reliable risk estimates. Current evidence does not warrant changes to vaccination recommendations; it instead supports careful injection technique, appropriate evaluation of persistent shoulder symptoms, and more specific adverse-event reporting.

### Limitations

As a narrative review, this study was not designed to provide the exhaustive coverage or reproducibility expected of a systematic or scoping review. No protocol was registered, and the search was limited to PubMed/MEDLINE, Google Scholar, and English-language publications. The restriction to English-language literature may have resulted in the omission of relevant case reports published in national or regional journals. Google Scholar results may vary over time and are not fully reproducible. Study selection and data extraction were not conducted within a prospectively defined systematic-review framework, and no formal risk-of-bias assessment was performed. Relevant unpublished observations or records not readily indexed may therefore have been missed.

## 5. Conclusions

Recombinant zoster vaccination represents a major advance in the prevention of HZ and its complications. Current evidence supports a favorable safety profile for Shingrix^®^, with expected local and systemic reactogenicity generally being transient and self-limiting. Evidence concerning cutaneous events is limited and heterogeneous and includes hypersensitivity reactions, vesicular or bullous eruptions, and episodes of wild-type VZV reactivation reported in temporal proximity to vaccination. Temporal proximity alone does not establish causality, and virological testing excluded vaccine-derived infection in the tested cases. Musculoskeletal events, including arthralgia and myalgia, were generally transient and self-limiting. Evidence concerning SIRVA is limited to a single case report following zoster vaccine administration in which the vaccine product was not specified. This isolated report does not permit estimation of incidence or attribution of SIRVA to Shingrix^®^. Current evidence does not justify changes to vaccination recommendations but supports proper injection technique, appropriate evaluation of persistent shoulder symptoms, and continued pharmacovigilance.

## Figures and Tables

**Table 1:** Comparison of the live-attenuated zoster vaccine (ZVL, Zostavax^®^) and the recombinant zoster vaccine (RZV, Shingrix^®^) according to formulation, target population, efficacy, reactogenicity, and evidence concerning Guillain–Barré syndrome.

Feature	ZVL (Zostavax^®^)	RZV (Shingrix^®^)
Type	Live-attenuated vaccine	Recombinant adjuvanted vaccine
Target population	Historically recommended for immunocompetent adults aged ≥60 years	Adults aged ≥50 years and adults aged ≥19 years who are or will be immunodeficient or immunosuppressed
ACIP recommendation	No longer available in the United States	Preferred zoster vaccine
Efficacy	Lower efficacy, decreasing with age and over time	More than 90% efficacy in pivotal clinical trials
Reactogenicity	Less frequent local and systemic reactions	More frequent but generally transient local and systemic reactions
GBS evidence	No comparable safety signal identified in the cited analyses	Small excess risk reported in adults aged ≥65 years; subsequent findings were not conclusive, and causality has not been established.

Abbreviations: ACIP, Advisory Committee on Immunization Practices; GBS, Guillain–Barré syndrome; RZV, recombinant zoster vaccine; ZVL, zoster vaccine live.

## Data Availability

The original contributions presented in this study are included in the article. Further inquiries can be directed to the corresponding authors.
